# Distributed implantation of a flexible microelectrode array for neural recording

**DOI:** 10.1038/s41378-022-00366-2

**Published:** 2022-05-12

**Authors:** Chunrong Wei, Yang Wang, Weihua Pei, Xinyong Han, Longnian Lin, Zhiduo Liu, Gege Ming, Ruru Chen, Pingping Wu, Xiaowei Yang, Li Zheng, Yijun Wang

**Affiliations:** 1 State Key Laboratory of Integrated Optoelectronics, Institute of Semiconductors, Chinese Academy of Sciences, 100083 Beijing, China; 2 University of Chinese Academy of Sciences, 100049 Beijing, China; 3 School of Future Technologies, University of Chinese Academy of Sciences, 100049 Beijing, China; 4 School of Microelectronics, University of Sciences and Technology of China, 230000 Hefei, China; 5 Institute of Automation, Chinese Academy of Sciences, 100190 Beijing, China; 6 Key Laboratory of Brain Functional Genomics, East China Normal University, 200062 Shanghai, China; 7 Brain Machine Fusion Intelligence Institute, 215131 Suzhou, China; 8 Technical Institute of Physics and Chemistry, Chinese Academy of Sciences, 100190 Beijing, China; 9 Chinese Institute for Brain Research, 102206 Beijing, China

**Keywords:** Electrical and electronic engineering, Other nanotechnology

## Abstract

Flexible multichannel electrode arrays (fMEAs) with multiple filaments can be flexibly implanted in various patterns. It is necessary to develop a method for implanting the fMEA in different locations and at various depths based on the recording demands. This study proposed a strategy for reducing the microelectrode volume with integrated packaging. An implantation system was developed specifically for semiautomatic distributed implantation. The feasibility and convenience of the fMEA and implantation platform were verified in rodents. The acute and chronic recording results provied the effectiveness of the packaging and implantation methods. These methods could provide a novel strategy for developing fMEAs with more filaments and recording sites to measure functional interactions across multiple brain regions.

## Introduction

Microelectrode arrays made of silicon or other stiff materials have to be implanted with the original topology^1–5^. A flexible multichannel electrode array (fMEA) with the same topology can be placed in the cortex at various positions within a certain range. The distance between the filaments and the depth of each filament can be adjusted based on the requirements^6,7^. Because of its flexibility, the fMEA can be implanted in various brain regions. We term this process distributed implantation. Direct implantation is infeasible due to the lack of required stiffness. Other materials or methods must be used to assist with fMEA implantation^8–13^. The injection is a promising implantation method. However, current injection technology requires complex preparation and a complicated packaging process after implantation, and the brain is vulnerable to overpressure^14–19^. Reinforced materials are popular for assisting with fMEA implantation. Degradable materials such as polyethylene glycol (PEG) or fibroin can be coated on the outside of the flexible filament to temporarily reinforce the stiffness^20–22^. After implantation, the coating material degrades and is absorbed by body tissue. However, in many cases, the stiffness of the reinforcing material is insufficient, which increases the difficulty of implantation. The reinforcing material needs to degrade and be absorbed, which may take time and result in toxicity^23,24^. Another popular method involves inserting an fMEA into the cortex with stiff shuttling probes or microneedles^7–10,25,26^. The fMEA filament is fixed on a shuttle probe^6,26,27^. However, there is usually more than one leg or filament on the fMEA^7,11^. In that case, complicated manual operations are required to individually align and fix the flexible shank on the shuttle probe^26^. Another option is to simultaneously align several filaments to the same number of shuttle probes and glue them together^7^. However, as the number of shanks increases, the current operational methods become incapable of implanting them effectively and safely. At present, neither the reinforced material nor the pre-glued shuttle probe satisfies the demands of fMEA distributed implantation. Elon Musk reported an implant known as Robert, which can automatically implant individual fMEA filaments^6^. However, Robert is too complex and expensive to be widely used.

An fMEA is composed of two parts: the implant part and the connector part. The recording sites are located at the front end of the implant part. The connector part, which is often soldered to a multichannel plug, remains outside and is mounted on the head. A bundle of wire lines connects the fMEA to the socket of a multichannel recording system. A microelectromechanical system (MEMS) process is commonly used to fabricate the fMEA. To minimize the volume of the implant part, the line width and line space of this part can be reduced to one micrometer or the nanometer scale^8,9,26,28,29^. However, the line spacing and dimension of the connector part must be increased to fit a plug connector^11,28^. A typical miniature plug connector, such as the Omnetics connector, a common interface for implant electrodes, has a minimum pin spacing of 640 μm. A 32 pin double-row Omnetics connector is 3 mm wide and 10 mm long. As the number of recording sites on the fMEA increases, the connector part can increase to an unreasonably large size^28^.

A potential solution for improving fMEAs was developed in this paper. To allow for simple and practicable implantation, homemade implanting tools, combined with the guide hole at the front end of the implant filaments, can semiautomatically catch and implant individual fMEA threads. The position and depth of each thread in the fMEA can be controlled with this tool. This reduces the manual operation while improving the implantation accuracy and speed. To reduce the fMEA volume necessary for plug-socket fan-out methods, we propose a direct interconnection method. The specially designed pads at the connector part of the fMEA, combined with the modified gold ball bonding process, connect the fMEA and the amplifier. This method eliminates the need for pairs of plug-socket connectors. The proposed implantation and interconnection methods provide a solution for developing fMEAs with multiple filaments and recording sites.

## Results

### Fabrication and packaging of the fMEA

**Fig. 1:**
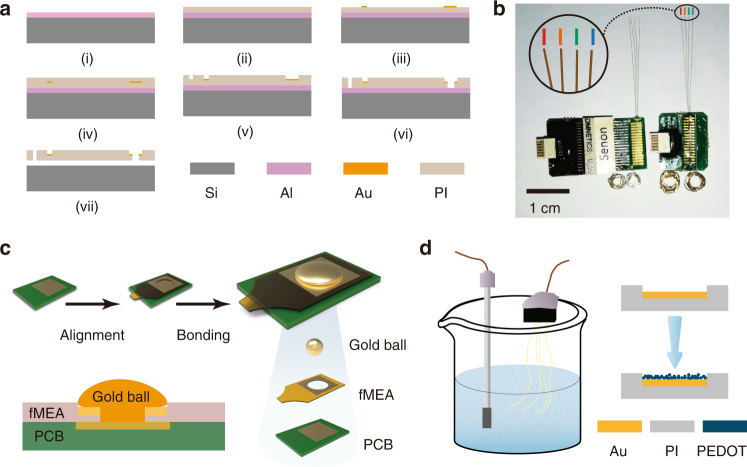
Preparation of flexible microelectrodes. **a** Schematic of the fMEA fabrication steps. (i) Deposition of the aluminum release layer. (ii) Spin-coating and curing of the bottom PI layer. (iii) Photolithographic patterning and growing of the gold layer. (iv) Spin-coating and curing of the top PI layer. (v) Photolithographic patterning and RIE to expose the recording sites and bonding pads. (vi) Photolithographic patterning and RIE to empty the implantation hole at the front end and the hollow welding points on the bonding pads. (vii) Release of the fMEA by aluminum etching. **b** Comparison photo of a conventional package with an Omnetics connector (left) and the integrated package (right). **c** Schematics of fMEA encapsulation by gold bonding. **d** The two-electrode system (left), with an fMEA as the working electrode and platinum(Pt) for the counter electrode and reference electrode. Schematic of electropolymerization of PEDOT (right)

The Polyimide (PI) 2600 series was chosen as the flexible substrate due to its excellent biocompatibility and flexibility. The device has a PI–metal–PI sandwich structure. The fabrication process of the fMEA is shown in Fig. 1a. The fMEA had four filaments and eight recording sites on each filament, as shown in Fig. 1b and Fig. S1. The filament was ~2.5 cm in length. Thus, it was long enough to be implanted in any area of the rat brain using the semiautomatic implantation platform. The width of the filament was only 70 μm to minimize the volume of the implant part. The recording sites were 10 μm long and arranged in a line along the filament. The distance between the recording sites was 100 μm. A loop at the front end of each filament was fabricated as a handle to assist with implantation. The loop was surrounded and marked by a gold ring to facilitate observation. The square bonding pad had a hollow round hole in the center to facilitate small-scale integrated packaging. To ensure that the filament had enough mechanical strength to withstand clamping, dragging, and pulling during the operation, polyimide with a tensile strength of 350 MPa was chosen. Flexible microelectrodes with a thickness of 6 μm were fabricated, and the bending stiffness of the thin-film filament was 1.07 × 10^−11^ Nm^2^ (Supplementary Methods). By adjusting the spin-coating parameters, an fMEA with a thickness of 2.4 μm or less can be produced.

Neural signal acquisition, amplification, digitization, and multiplexing were executed by the amplifier module to ensure signal quality and reduce the number of external leads. We connected the fMEA to the module with a modified gold ball bonding process, as shown in Fig. 1c. The volume of the module was less than half of the volume of a traditional package microelectrode with an Omnetics connector, as shown in Fig. 1b. The proposed fMEA with the amplifier weighs 0.580 g, while the traditional package microelectrode weighs 1.328 g. The small-size integrated packaging solution based on gold ball bonding is easy to operate and can be scaled up to hundreds or more channels.

### Impedance test and microtopography observation

**Fig. 2:**
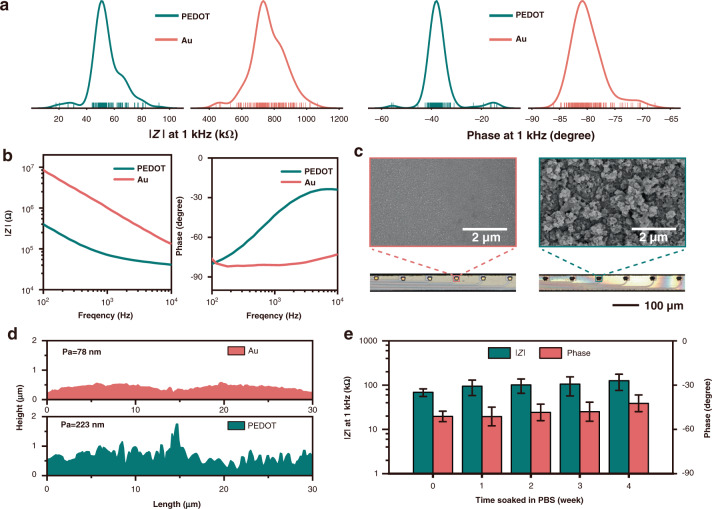
Evaluation of flexible microelectrodes in vitro. **a** Distribution of the representative impedances measured at 1 kHz for the gold and PEDOT surfaces (*n* > 160). **b** Representative impedance spectroscopy over a frequency range of 10,000 Hz. **c** Representative SEM images of the gold (left) and PEDOT (right) surfaces (upper) and representative CLSM images of the gold (left) and PEDOT (right) microelectrodes (lower). **d** Representative distribution of the surface height at the recording sites. **e** The impedance of two PEDOT microelectrodes (m6 and m7) at 1 kHz. Microelectrodes were soaked in PBS for 4 weeks, and the error bars indicate the standard error

Poly(3,4-ethylenedioxythiophene) (PEDOT) is commonly used to modify recording sites to improve impedance and stability^30–33^. PEDOT was grown on the recording sites by galvanostatic polymerization with an aqueous solution containing 0.02 M EDOT monomer and 0.1 M TsONa (sodium p-toluene sulfonate) electrolyte, as shown in Fig. 1d. The impedance data were collected from more than 160 channels, as shown in Fig. 2a. Electrochemical impedance spectroscopy was measured in the PBS solution before and after electroplating, as shown in Fig. 2b. The interface impedance decreased by an order of magnitude after electroplating. Confocal laser scanning microscope (CLSM) observations showed that the recording interface changed from yellow to darker in color after electroplating, as shown in Fig. 2c. The surface morphology and microstructure of the recording pads observed with scanning electron microscopy (SEM) are shown in Fig. 2c. The gold pad was flat, while the PEDOT pad was rough. The average roughness of the gold surface was ~78 nm, while the corresponding value of the PEDOT surface was ~233 nm, as measured with CLSM, and the results are shown in Fig. 2d. The roughness of the PEDOT surface, combined with the surface activity, reduced the electrochemical impedance.

The impedance stability of the fMEA is shown in Fig. 2e. The impedance increased from the initial value of 68.8 ± 13.5 kΩ (mean ± s.e.m.) to 94.2 ± 35.9 kΩ after one week of immersion. Over the next three weeks, the average impedance value was stable. The average impedance increased to ~126.3 ± 24.8 kΩ in the fourth week. The impedance evolution in vitro was consistent with a previous report^34^. The increased impedance may be caused by electrode surface absorption.

### Platform construction and implantation test

**Fig. 3:**
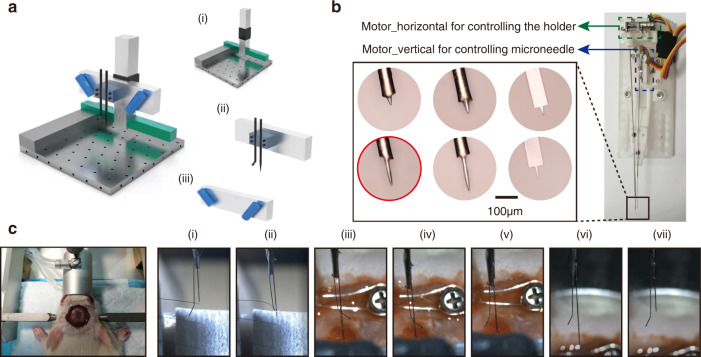
Implantation of flexible microelectrodes. **a** Schematic of the semiautomated implantation platform. The platform has three parts: (i) the triaxial positioning system, (ii) the filament pick-up module, and (iii) the observation system. **b** Construction of the filament pick-up module for handling the flexible filaments. The green dashed box indicates “motor_ horizontal”, which rotates the holder, and the blue dashed box indicates “motor_vertical”, which moves the microneedle up and down. Multiple microneedles are shown in the black box at the lower-left corner, and the stepped microwire used during implantation is indicated by the red circle. **c** Images of rat surgery and time sequence photographs of implantation. (i) Aligning the microneedle with the implantation hole by moving the triaxial positioning axis. (ii) Inserting the microneedle into the implantation tool and picking up the filament by controlling the motors. (iii) Moving the triaxial positioning axis until the microneedle contacts the target brain surface. (iv) Moving the triaxial positioning axis down to the target area. (v) Rotating the holder and releasing the filament. (vi) Moving the triaxial positioning axis to remove the microneedle. (vii) Moving the microneedle to ~2 mm above the holder

We built a semiautomated implantation platform for the distributed implantation of the flexible microelectrodes. The implantation platform was composed of three subsystems, as shown in Fig. 3a and Fig. S2, including the triaxial positioning system, the filament pick-up and insertion module, and the observation system. The filament pick-up and insertion module and the observation system were mounted on the triaxial positioning system and can be moved and positioned precisely. The triaxial positioning system was computer-controlled. The resolution of all axes was 1 μm. The travel lengths of the X, Y, and Z axes were 300, 300, and 100 mm, respectively. As shown in Fig. 3b, the filament pick-up and insertion system was composed of a rigid microneedle and a holder. The microneedle tip was shaped like a T to hook the filament before implantation and to remove it after implantation. The microneedle moved vertically, powered by a motor (motor_vertical), to adjust its relative position with the holder during implantation. The holder was *L*-shaped to allow the microneedle to control the implanted filament. The holder was driven by a motor (motor_horizontal) and rotated slightly to lean on or away from the microneedle. The observation system contained two microscopes that were placed on both sides of the filament pick-up and insertion system, aimed at the microneedle tip at an ~45° angle. The implant position can be flexibly selected with the observation system to effectively avoid cerebral facial blood vessels.

The implantation operations can be easily accomplished with the semiautomated implantation platform, as shown in Fig. 3c. The filament was picked up and moved until it came into contact with the cortex surface. Then, the filament was implanted vertically at a rate of ~50 μm per second. As the filament approached the target area, the implantation rate was reduced to 10 μm per second. In 22 implantation operations, all picking up, transferring, and location motions were performed with micron-level accuracy. Twenty filaments were successfully implanted. There were two cases of implantation failure due to failure to separate the filament from the implant microneedle. The average pick-up and location time of each filament was less than 1 min, and the success rate reached 90%.

### Electrophysiological recording

**Fig. 4:**
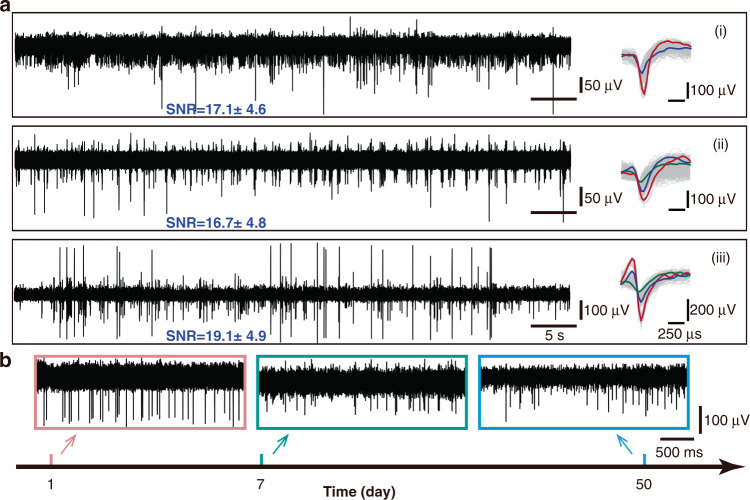
Extracellular signals in rats. **a** Neural signals acquired from an fMEA implanted in the rat hippocampal CA1 region after 500 Hz high-pass filtering. **b** Neural signals recorded from an fMEA (m1) on the 1st, 7th, and 50th day after implantation, respectively

Neurostudio was used to acquire neural signals at a sampling rate of 30 kHz, and MATLAB was used for data processing. Five microelectrodes (m1–m5) were implanted in five rats. Microelectrodes m1 and m2 were implanted in the hippocampus CA1 region, while microelectrodes (m3–m5) were implanted in both the CPu (caudate putamen) and PrL (prelimbic cortex) regions. Unfortunately, microelectrode m5 had to be discarded because the rat died of hypothermia after surgery. The neural signals of the rats were recorded after microelectrode implantation, as shown in Fig. 4 and Fig. S3. The spiking yield was 43.75 ± 0.09% (mean ± s.e.m.) across the four effective surgeries, which is comparable to the results reported by Neuralink (45.60 ± 0.03%)^6^. More than one neuron can be sorted from one channel. The extracellular signals were acquired, and three typical channels are shown in Fig. 4a. The signal-to-noise ratio (SNR) reached as high as 19 dB. We recorded the extracellular signals for 7 weeks to assess the long-term stability of the microelectrode in vivo, as shown in Fig. 4b. The amplitude was reduced after 7 weeks. However, the spikes were still clearly distinguishable.

## Discussion

Flexible microelectrodes are expected to contribute to brain research due to their low mechanical stiffness, which promotes tissue compatibility and improves the stability of long-term recordings^35–38^. Unlike traditional rigid microelectrodes, flexible microelectrodes can be flexibly arranged to achieve on-demand implantation in the whole brain, which is suitable for various electrophysiological experiments, such as the simultaneous collection of neural signals from multiple brain regions.

A semiautomated flexible microelectrode implantation system was developed to implant fMEAs. The fMEA does not require a laborious manual arrangement, and the implantation process is simple and quick. Each filament was implanted separately, and the implant position was flexibly selected to avoid cerebral facial blood vessels. This implantation method can be repeated and scaled up to implant flexible microelectrodes with hundreds or thousands of channels. When compared with the highly integrated surgical robot built by Neuralink, our semiautomated system has several shortcomings in terms of functionality, but it provides a simple yet reliable platform for flexible filament implantation in general research.

However, the bulky packaging volume of conventional Omnetics connectors is unacceptable as the number of flexible microelectrode channels increases. This becomes a non-negligible problem when packaging high-throughput flexible microelectrodes with small sizes. The proposed gold ball bonding method directly integrated the fMEA and the chip, minimizing the size and weight of the device. The chip was integrated with the fMEA, allowing the entire system to function with only a few power lines and data lines. The small-size integrated packaging solution is easy to scale up to more channels.

## Materials and methods

### Preparation of the fMEA

As shown in Fig. 1a, the key fabrication steps are as follows: (i) A 4 inch, 250 μm-thick single-polished silicon wafer was selected as the substrate and cleaned using piranha solution. A 100 nm-thick aluminum layer was deposited on the substrate by sputtering to serve as the release layer. (ii) The bottom PI layer was spin-coated and cured. (iii) A 150 nm-thick patterned gold layer was grown by vacuum evaporation and patterned by a lift-off process. (iv) After the top PI layer was spin-coated and cured, thermal annealing was conducted at 350 °C for 1 h in a nitrogen atmosphere to improve the stability of the microelectrode. (v) The wafer was patterned by photolithography. The top PI layer was then etched by reactive ion etching (RIE) to expose the recording sites at the front end and the bonding pads at the rear end. (vi) The shape of the fMEA was patterned and etched with the same methods as in the previous step. (vii) The flexible microelectrodes were released from the silicon substrate by aluminum etching and cleaned with deionized water.

### Connecting the fMEA to the amplifier module

The fMEA and the amplifier module were connected directly without a plug and socket. The module was custom-made based on a 32-channel application-specific integrated circuit (ASIC)^29^. We connected the fMEA to the module with a modified gold ball bonding process, as shown in Fig. 1c. The connection pads at the rear of the fMEA were hollow in the center. The position and arrangement of the fMEA pads were the same as those of the pads on the amplifier module. After the alignment, the pads of the fMEA were aligned with the corresponding pads on the circuit. The fMEA margin was glued to the circuit for temporary fixation. The ball size and the press were adjusted before bonding to ensure that the gold ball and the pads under it were tightly fixed. The optimal gold ball size in this study was 100 μm in diameter. The press was 2 N. Medium ultrasonic power was adopted. The gold ball was welded on the circuit pad through the hollow hole (60 μm in diameter) of the fMEA, and the peripheral region of the ball was tightly pressed to the gold pad of the fMEA. With this method, the fMEA and the module were connected. Epoxy or wax was used to protect and reinforce the bonding area. The module-integrated fMEA is shown in Fig. 1b.

### Electrodeposition of PEDOT

PEDOT was grown on the recording sites by galvanostatic polymerization with an aqueous solution. The electroplating solution was prepared by ultrasonically dispersing 0.02 M EDOT in a 0.1 M sodium p-toluene sulfonate electrolyte. A 4 mm square platinum foil electrode was used as both the counter electrode and reference electrode to construct a two-electrode system, as shown in Fig. 1d. The electrochemical deposition was performed with the two-electrode system. An electrochemical workstation was used to supply a constant current of 6.5 nA for 15 s to all recording sites.

### Impedance measurement

The impedance test was performed in the phosphate-buffered saline (PBS) solution discussed in the electrochemical deposition section. The impedance measurements were performed by applying a sinusoidal AC voltage of 10 mV at 1 kHz. The electrochemical impedance spectroscopy measurements were obtained by applying a 10 mV sinusoidal AC voltage. The frequency ranged from 100 to 10,000 Hz. To assess the impedance stability, the fMEA was immersed in a 36 °C PBS solution, and the impedance was measured at 1 kHz once every 24 h for 4 weeks.

### Animal surgery

Adult Sprague–Dawley female rats (200–220 g) were used in this paper. All procedures were approved by the Animal Advisory Committee at East China Normal University and were performed in accordance with the National Institutes of Health Guidelines for the Care and Use of Laboratory Animals. The rats were anesthetized with isoflurane (1–5%) and placed in the stereotactic apparatus. A 37 °C constant heating blanket was spread under the animal. Erythromycin ointment was applied to the eye to prevent dehydration. The skull was exposed with a small incision to determine the target location. A 3 mm diameter hole was created with a craniotomy. The dura was resected, and brain tissue was maintained under artificial cerebrospinal fluid to prevent desiccation.

### Microelectrode implantation

The stereotactic apparatus with a craniotomy rat was transferred and fixed on the implantation system. Before implantation, a stool with the packaged fMEA was placed on the rat’s neck, and the fMEA was connected to the Neurostudio system to record the extracellular signal. The tips of the four filaments stretched ~1 mm beyond the table facet. The implantation process can be divided into three steps. The key operations are shown in Fig. 3c and described as follows.

The filament pick-up and insertion module were set to their initial state. The holder was kept away from the microneedle and at least 2 mm below it. By manipulating the triaxial positioning system, the microneedle tip was moved to the vicinity of the loop of the first filament. Under microscopy observation, the microneedle was aligned with and inserted into the loop. Motor_vertical was used to move the microneedle down to ~0.2 mm below the holder. The holder was rotated slightly to lean on the microneedle with motor_horizontal. The filament was hooked onto the tip of the microneedle and clamped between the holder and the microneedle rod. The filament was picked up and moved with the needle.

By manipulating the triaxial positioning system, the filament was moved to a position directly above the operating area on the rat’s head. A location without blood vessels was chosen, and the position of the microneedle was adjusted until the tip touched the cortex surface; this position was set as zero depth. The filament was implanted by manipulating the *Z*-axis of the positioning system at a rate of ~50 μm per second. When the electrode approached the target area, the rate was decreased to 10 μm per second. The extracellular signal and position coordinates were monitored during implantation.

After the filament reached the target location, the holder was rotated to loosen the filament. Using the triaxial positioning system, the microneedle was withdrawn ~200 μm to separate it from the loop at the front end of the filament. The filament was released from the pick-up and insertion module and remained in the brain. After this operation, the pick-up and insertion module was reset to its initial state. All filaments were implanted by repeating the above steps.

All filaments of microelectrodes m1 and m2 were successfully implanted into the hippocampal CA1 region. Microelectrodes m3–m5 were successfully implanted into both the CPu and PrL regions. Two filaments (marked with red and orange in Fig. 1b) of the microelectrode were implanted into the CPu region, while the other two filaments (marked with green and blue in Fig. 1b) were implanted into the PrL region. Microelectrode m5 was discarded because the rat died of hypothermia after surgery.

### Acute and chronic electrophysiological acquisition

After all of the filaments were implanted, the filaments were fixed to the rat skull with dental acrylic. The connector part of the fMEA was carefully transferred from the stool to the rat head and fixed with dental acrylic. Acute neural signals were recorded by Neurostudio on the day of surgery. The rat was then released. After it recovered from anesthesia, it was raised in a separate cage. One week after implantation, the neural activity was collected while the rat was awake or sleeping in a box. Seven weeks after surgery, extracellular signals were collected in the free-moving state.

## Supplementary information

Supplemental Material
