# Supplementary material for: Distributed implantation of a flexible microelectrode array for neural recording
Source: Microsyst Nanoeng. 2022 May 12;8:50. doi: 10.1038/s41378-022-00366-2 (PMC9098495; doi:10.1038/s41378-022-00366-2)
Supplement: Supplementary file 1 — Supplemental Material [file 41378_2022_366_MOESM1_ESM.docx]

Supplementary Information

Distributed Implantation of a Flexible Microelectrode Array for Neural Recording

Chunrong Wei^1,2,3^, Yang Wang^1,4^, Weihua Pei^1,2*^, Xinyong Han^5^, Longnian Lin^6^, Zhiduo Liu^1,2^, Gege Ming^1,2,3^, Ruru Chen^7^, Pingping Wu^2,3,8^, Xiaowei Yang^1^, Li Zheng^1,2,3^and Yijun Wang^1,2,9^

^1^ State Key Laboratory of Integrated Optoelectronics, Institute of Semiconductors, Chinese Academy of Sciences, Beijing 100083, China.

^2^ University of Chinese Academy of Sciences, Beijing 100049, China.

^3^ School of Future Technologies, University of Chinese Academy of Sciences, Beijing 100049, China.

^4^ School of Microelectronics, University of Sciences and Technology of China, Hefei 230000, China.

^5^ Institute of Automation, Chinese Academy of Sciences, Beijing, 100190, China.

^6^ Key Laboratory of Brain Functional Genomics, East China Normal University, Shanghai 200062, China.

^7^ Brain Machine Fusion Intelligence Institute, Suzhou 215131, China.

^8^ Technical Institute of Physics and Chemistry, Chinese Academy of Sciences, Beijing 100190, China.

^9^ CAS Center for Excellence in Brain Science and Intelligence Technology, Shanghai 200031, China.

* Corresponding authors: Weihua Pei (e-mail: peiwh@semi.ac.cn)

**Supplementary Methods**

**Calculation of the bending stiffness for neural microelectrodes.**

The bending stiffness *K* is used to evaluate the mechanical characteristics of the tissue-electrode interface. For a planar thin-film probe, *K* can be calculated from^S1^:

$$K=E_{s}\frac{wh^{3}}{12}$$

where *Es* is Young’s modulus of the polyimide, *h* and *w* are the total thickness and width of the probe, respectively. When *E_s_* = 8.5 Gpa, *h* = 6 μm, *w* = 70 μm. According to the formula, the bending stiffness of the filament is 10.71×10^-12^ Nm^2^.

**Supplementary References**

S1. Steif, P. S. Mechanics of materials. (Pearson: Upper Saddle River, NJ, 2012).


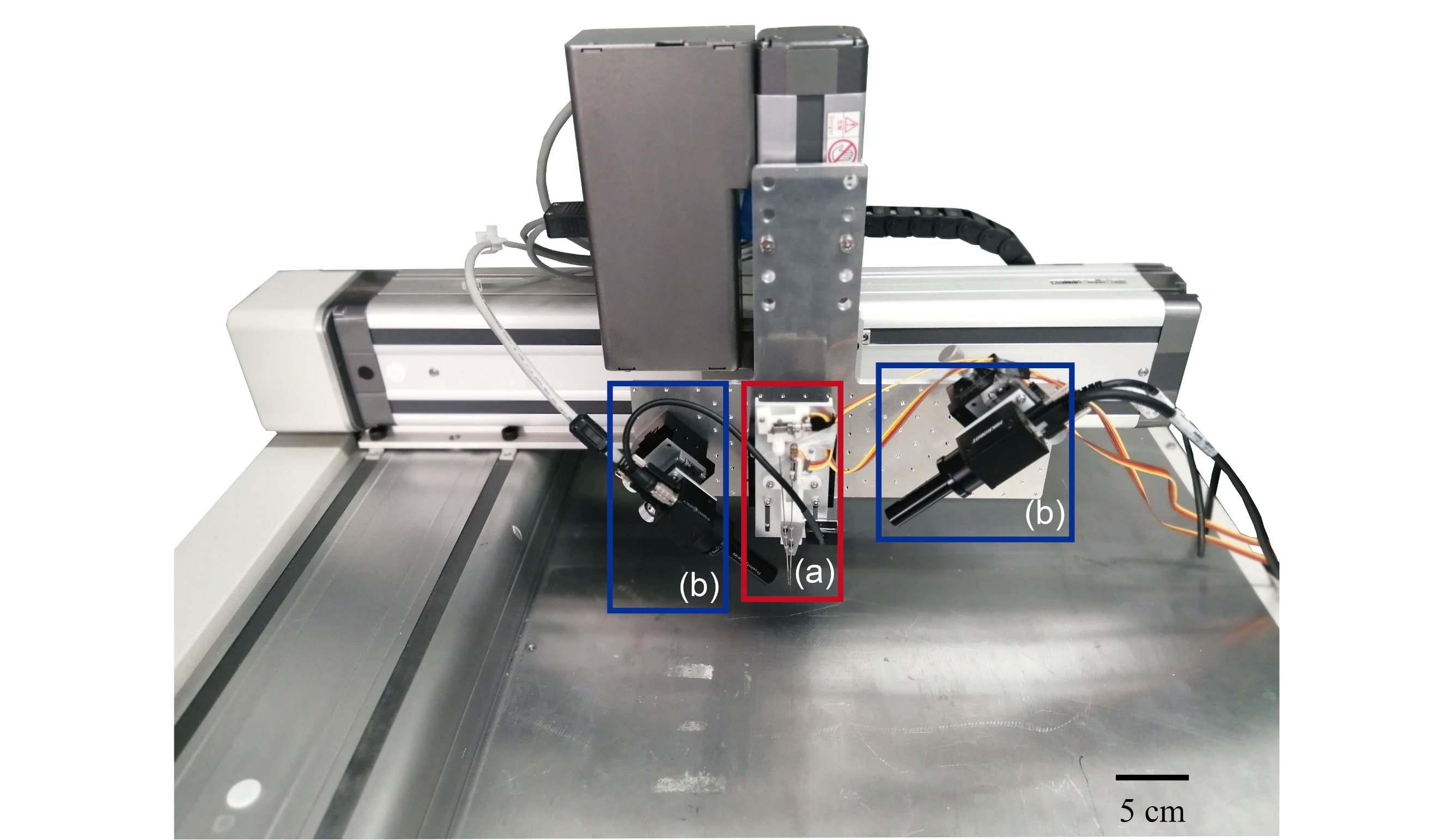


Fig. S2 Image of the semi-automated implantation platform. The platform consists of the triaxial positioning system mounted with (a) the filament pick-up and insert module and (b) the observation system.


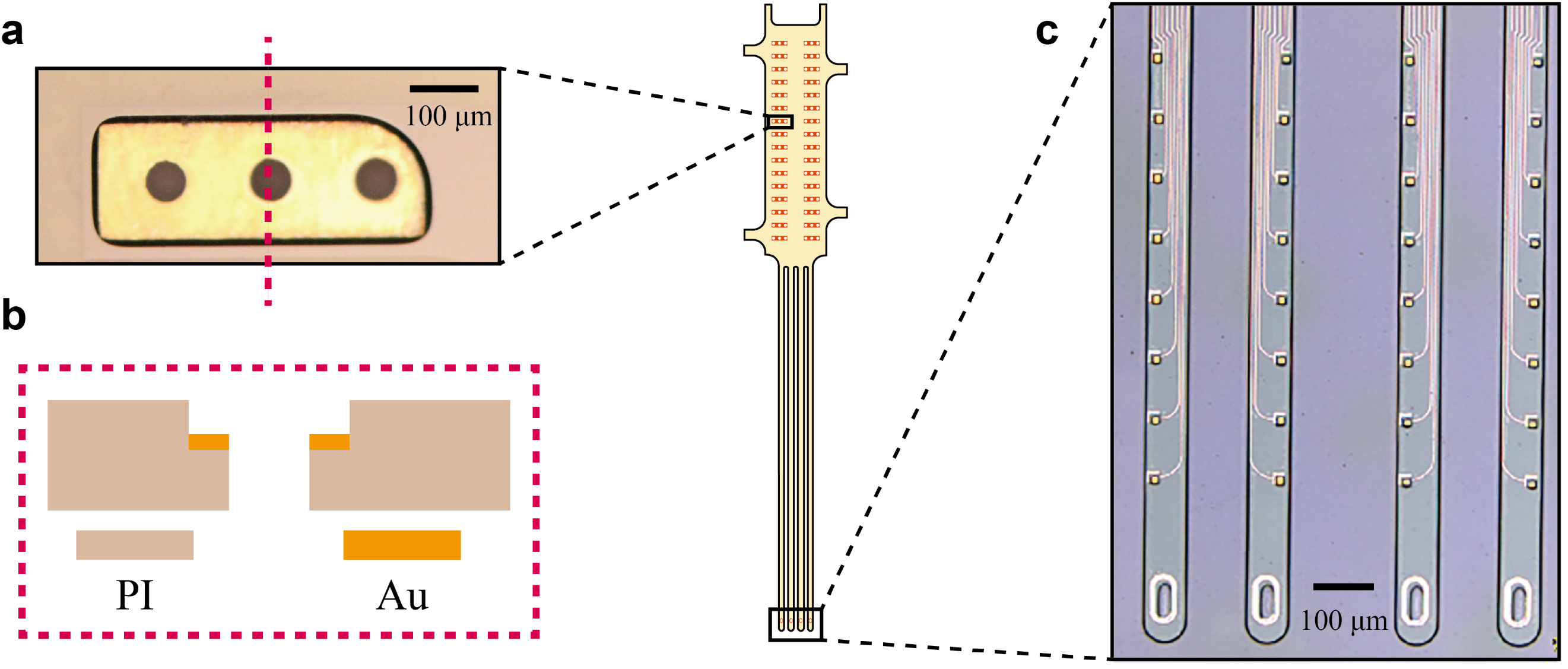


Fig. S1 Schematic of the fMEA. (a) Image of the bonding pad. (b) Cross-sectional image at the red dashed line in (a). (c) Image of recording sites and implantation loops.


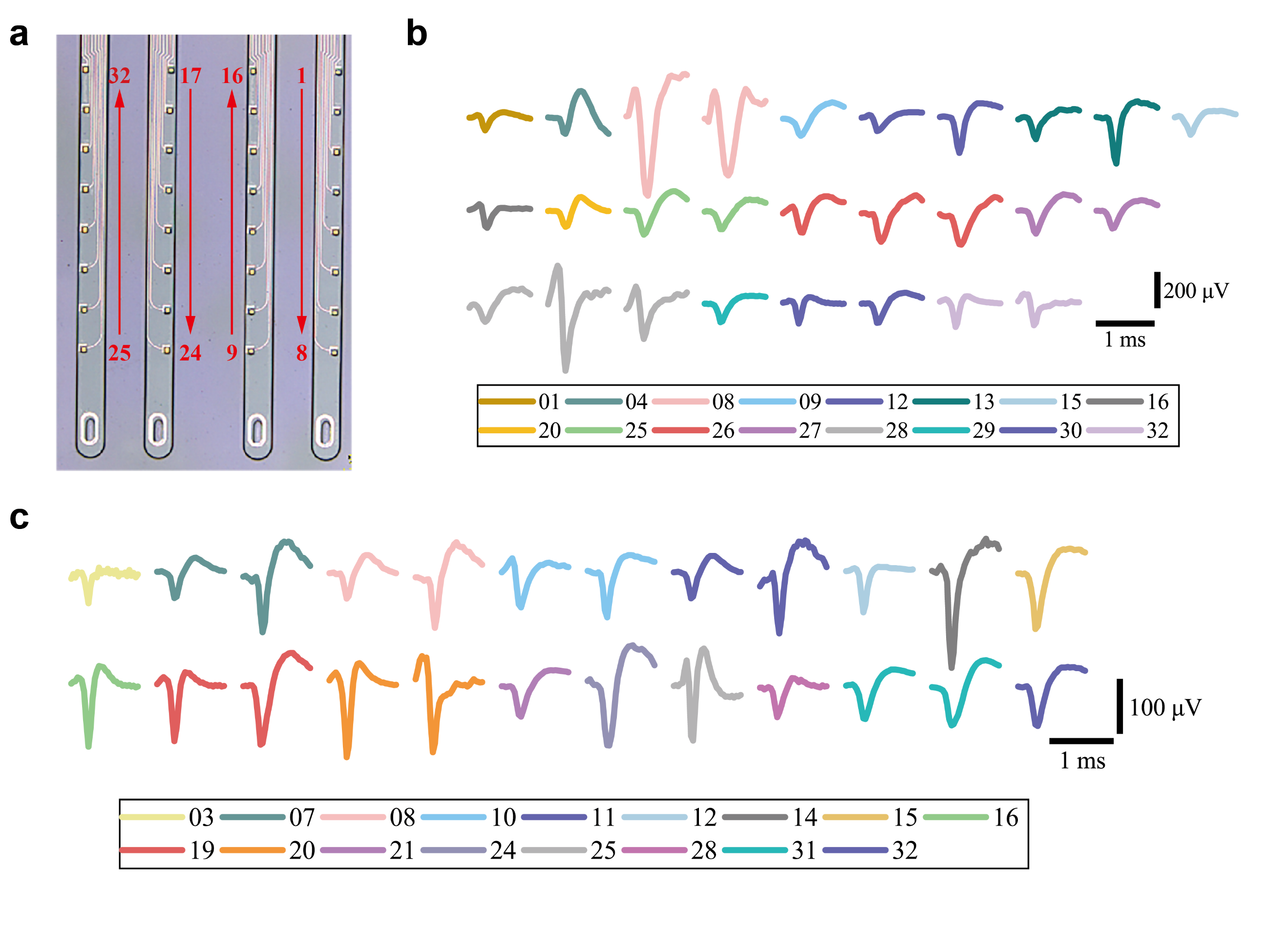


Fig. S3 (a) Recording sites and channels number. (b) Spike waveforms recorded from the microelectrode (m1) implanted in hippocampus CA1 of rat, and numbers indicate channel location on microelectrode. (c) Spike waveforms recorded from the microelectrode (m4) implanted in both the CPu (channel 1-16) and PrL (channel 17-32) of rat, and numbers indicate channel location on microelectrode.
